# Nontoxic Medical Imaging Agents Form Toxic DBPs

**DOI:** 10.1289/ehp.119-a511

**Published:** 2011-12-01

**Authors:** Carol Potera

**Affiliations:** Carol Potera, based in Montana, has written for *EHP* since 1996. She also writes for *Microbe*, *Genetic Engineering News*, and the *American Journal of Nursing*.

Iodinated X-ray contrast media (ICM), used medically to highlight soft tissues, are considered generally safe, and patients excrete 95% of the dose within 24 hours, making them a valuable tool in medical imaging. But a new study shows that excreted ICM react with chloramine and chlorine at water treatment plants to form toxic iodinated disinfection by-products (iodo-DBPs).^1^ “ICM are definitely a source of iodo-DBPs in drinking water,” says study coauthor Susan Richardson, a research chemist at the U.S. Environmental Protection Agency (EPA) in Athens, Georgia, although she says natural iodide salts from saltwater intrusion are probably the major source of these contaminants.

In an earlier study of drinking water collected from 23 cities, Richardson and toxicologist Michael Plewa of the University of Illinois at Urbana–Champaign detected iodo-acids and iodo-trihalomethanes, both highly toxic iodo-DBPs.^2^ They assumed that the source of iodine was natural iodide salts from intrusion of ocean water or natural minerals in the source water. Yet surprisingly, some samples had high concentrations of iodo-DBPs despite little or no detectable natural iodide in the water. Their new study, done in collaboration with Stephen Duirk at the University of Akron and Thomas Ternes at the German Federal Institute of Hydrology, suggests that ICM, which contain three iodines per molecule, were an unexpected source of iodine in 10 of the original 23 cities. Further laboratory tests confirmed that ICM form iodo-DBPs in the presence of chlorine and chloramine, which are commonly used to treat drinking water.^1^

ICM are nearly impossible to remove from water by conventional treatment methods, says environmental engineer David Sedlak, codirector of the Berkeley Water Center at the University of California, Berkeley, and the compounds end up in rivers used for drinking water.^3^ Richardson’s team screened drinking water for five common ICM (io-pamidol, iopromide, diatrizoate, iomeprol, and iohexol). Iopamidol was detected in the highest concentrations—up to 2,700 ng/L, compared with less than 100 ng/L for the majority of the other ICM—and was chosen as the test ICM for controlled experiments. When iopamidol was dissolved in river water spiked with chlorine or chloramine, high levels of iodoacetic acid and iodo-trihalomethanes were produced in a dose-dependent manner. In comparison, no iodo-DBPs formed in the absence of iopamidol in river water containing the two disinfectants. Iodo-DBPs form best at pH 6.5–8.5, conditions common at water treatment plants.^1^

ICM are a major medical breakthrough—“We don’t want to stop using them,” Richardson says. Other types of ICM may form lower concentrations of iodo-DBPs than io-pamidol, and Richardson will test this hypothesis and seek insight into how ICM interact with chlorinated disinfectants. Better understanding of the reactions involved could ultimately prevent toxic by-products.

More than 600 DBPs are known, and Plewa’s laboratory has systematically analyzed about 80 for genotoxicity and cytotoxicity in mammalian cells. Iodo-DBPs are more toxic than brominated and chlorinated analogs, and iodoacetic acid is the most genotoxic of all DBPs tested so far.^4^ Iodo-DBPs are being investigated for links to bladder cancer and birth defects.^5^^,^^6^ The EPA regulates some chlorinated and brominated DBPs in drinking water but not iodo-DBPs, whose toxicity is only just being recognized.^7^

“We don’t think about disinfection of drinking water modifying water contaminants and generating enhanced toxicity,” Plewa says. In this case, he says, “A pharmaceutical with absolutely no genotoxicity [i.e., ICM] forms highly genotoxic iodo-DBPs. How many other reactions with exogenous agents occur when water is disinfected for drinking?” Plewa points out that this toxic transformation of ICM differs from the impact of other pharmaceutical contaminants in water, such as estrogenic hormones, which directly cause adverse responses.

It’s been assumed that the presence of ICM in drinking water poses no human health concerns. “But when ICM are transformed into compounds that are more toxic than the parent compound, it raises questions about assessing the potential risks associated with discharging these compounds into the environment,” Sedlak says. His research shows that ICM in wastewater are largely found near hospitals and imaging laboratories.^8^ “It would be straight-forward to collect and treat these compounds at the source,” Sedlak says. Plewa offers a practical solution: “Patients given ICM could urinate before leaving hospitals, and the collected urine could be safely disposed of at the hospital.”
